# A Novel, Cell-Free Therapy to Enter Our Hearts: The Potential Role of Small EVs in Prevention and Treatment of CVD

**DOI:** 10.3390/ijms23073662

**Published:** 2022-03-27

**Authors:** Ioanna Lazana, Constantinos Anagnostopoulos

**Affiliations:** 1 King’s College Hospital NHS Foundation Trust, London SE5 9RS, UK; 2 Cell and Gene Therapy Laboratory, Biomedical Research Foundation of the Academy of Athens, 115 27 Athens, Greece; 3 Onassis Cardiac Surgery Centre, 176 74 Athens, Greece; constant_anag@yahoo.co.uk

**Keywords:** extracellular vesicles, cardiovascular disease, ischemia-reperfusion injury

## Abstract

Heart disease constitutes one of the leading causes of morbidity and mortality worldwide. Current therapeutic techniques, such as interventional revascularization, although lifesaving, come along with myocardial injury related to the reperfusion itself, called ischemia-reperfusion injury, which is an added factor for increased morbidity. For that reason, there is an imperative need for novel therapies to be developed that would either prevent or treat myocardial injury. Extracellular vesicles (EVs), specifically small EVs (sEVs), have proven to be important mediators of intercellular communication. The fact that they carry information reflecting that of the parental cell makes them an ideal candidate for diagnostic purposes. sEVs derived from immunoregulatory cells, such as mesenchymal stem cells or cardiac progenitor cells, could also be used therapeutically to exert the primary immunomodulatory function but without carrying the side effects related to cell therapy. Furthermore, as a natural product, they have the added advantage of low immunogenicity, offering the potential for safe drug delivery. In the field of cardiology, there has been great interest in the therapeutic and diagnostic potential of sEVs with significant translational potential. Here, we review the potential use of sEVs in the context of myocardial ischemia and ischemia-reperfusion injury.

## 1. Introduction

Cardiovascular diseases (CVDs) constitute one of the leading causes of morbidity and mortality worldwide [1]. Current therapies involve the use of pharmacological agents, such as anticoagulants and antiplatelets, and interventional therapies, such as primary coronary intervention (PCI) and coronary artery bypass surgery (CABG). Despite the progress in such therapies in recent years, one-year mortality after ST-elevation myocardial infarction (STEMI) has failed to improve [2], as they have little effect on the injured myocardium or in slowing down the progression of damage. The duration of myocardial ischemia (MI) has also been shown to be a crucial determinant affecting long-term outcomes, so there has been great effort by cardiac centers to minimize the time from chest pain to therapeutic intervention. However, reperfusion treatments, such as PCI, which have been shown to be associated with reduced infarct size and improved left ventricular systolic function (LVSF), come (paradoxically) at a price [3]. It is now well established that restoration of blood flow itself after MI leads to accelerated myocardial apoptosis and subsequent (irreversible) myocardial tissue loss, known as ischemia-reperfusion injury (IRI) [4]. Although many pathways and molecules have been shown to be involved, the exact mechanism of IRI remains to be elucidated [5]. It is of particular importance that IRI itself may contribute up to 40% of total infract size post-MI [6], emphasizing the imperative need to balance the disparity between restoration of blood flow and the related injury. Unfortunately, the development of therapeutic strategies to control IRI has been disappointing. The first non-pharmacological intervention included the application of ‘ischemic pre-conditioning’ [7], which, despite the encouraging pre-clinical results, was hard to implement due to the unpredictability of coronary artery occlusion. The next breakthrough was the application of ‘ischemic post-conditioning’, which involved brief alterations of ischemia and reperfusion immediately after flow restoration [8]. Initial results revealed a 30–40% reduction in infarct size, but unfortunately, it failed to improve outcomes in a subsequent randomized controlled trial [9]. Various pharmacological drugs have also been trialed in recent decades, such as cyclosporin A [10], metoprolol [11], abciximab [12], etc. Again, despite the originally encouraging results, subsequent larger studies failed to show a survival benefit. For that reason, new therapies are required to attenuate IRI, as protection from IRI would minimize infract size and improve patient prognosis. There is subsequently an imperative need for novel therapies to be developed that would either preserve the functional myocardium or repair the damage.

Extracellular vesicles (EVs), particularly small EVs (sEVs), have emerged as important mediators of intercellular communication. All cells in the human body are able to produce sEVs in an effort to communicate or exchange material/information not only with neighboring cells but also with distant tissues [13]. More interestingly, the function of sEVs reflects that of the parental cell, offering a huge potential as carriers of immunomodulatory messages.

## 2. sEV Production and Function

sEVs can be broadly divided into apoptotic bodies, microvesicles, and sEVs (previously termed exosomes). sEVs are nanosized (30-120nm) particles consisting of a lipid bilayer membrane and containing lipids, genetic material (mainly miRNA and mRNA), and proteins [13]. They form by internal budding of the plasma membrane and formation of early sorting endosomes (ESE) by merging of primary endocytic vesicles. ESE then progresses to generate multivesicular bodies (MVBs) via endosomal sorting complex required for transport (ESCRT), which is located on the cytoplasmic side of the endosome [14]. The MVBs then move towards the cell membrane, with which they fuse and get released into the extracellular space [15]. The sEV release can be either constitutive or inducible. In the first case, the process is controlled by RAB, GTPases, glycosphingolipids, WNT5A, and G protein [16], whereas in the latter, sEV release is controlled by stress factors and signaling, such as hypoxia, heat shock, DNA damage, calcium release, and lipopolysaccharides [17]. The uptake of sEVs by the recipient cell is well described and involves various processes, such as interaction with membrane receptors, membrane fusion, and endocytosis [18].

sEVs have attracted significant attention recently, owing to their unique characteristics, rendering them an ideal candidate for both diagnostic and therapeutic purposes. These characteristics include: (i) their cargo is highly regulated, reflecting that of the parental cell; (ii) they lack a nucleus, so they cannot proliferate/differentiate in vivo and have no metabolic potential; (iii) they are a natural product and carry low/no risk of mutagenesis or tumor formation; (iv) they are nanosized, so they can cross natural barriers and reach distant targets; (v) the lipid bilayer protects their cargo from degradation; (vi) depending on the parental cell, they may confer low immunogenicity [19,20]; and (vi) they can be stored at −80 °C safely, without any alteration in their biological properties [21]. In view of these properties, there has been an increasing interest in their role and contribution in pathogenesis, prevention, and treatment of many diseases, including heart disease. Here, we summarize the potential use of sEVs in the context of coronary syndromes and ischemia-reperfusion injury (Table 1).

## 3. Role of sEVs in the Treatment of Myocardial Infraction and IRI

Mesenchymal stem cells (MSCs) have long been used for the treatment of myocardial ischemia, in view of their low immunogenicity, multidirectional differentiation, and immunoregulatory properties, in an effort to preserve the functionality of the myocardium [38]. However, despite the originally positive results, not all studies have shown a positive impact, owing to the reduced viability of transplanted MSCs and the related side effects (arrhythmias, myocardial hypertrophy, and carcinogenesis), [22]. It is now well established that the therapeutic effects of MSCs are (at least partially) mediated by paracrine factors [39]. This evidence was supported by Timmers et al. [39], who used human embryonic MSC-derived culture medium (hESC-CM) to study the potential impact on infract size. Intravenous and intracoronary administration of hESC-CM led to a significant reduction in the size of infract, which was associated with improved hemodynamic parameters and cardiac function (systolic and diastolic) in general. Taking this further, a reduction in oxidative stress and cell apoptosis and modulation of transforming growth factor-beta (TGF-β) singling were found to be responsible for the aforementioned protective effect. Most importantly, the cardioprotective factors were proven to be in the >1000-kDa fraction, with size ranging from 100 nm to 220 nm, which is within the size range of sEVs (or at least part of it).

Based on the above, alternative options to cell therapy have been sought, focusing on sEVs, with very encouraging results [23,40]. Xu et al. [24] explored MSCs as a potential source of sEVs. Using different sources of MSCs (adipose tissue, bone marrow, and umbilical cord blood) to isolate sEVs, the authors were able to demonstrate a significant reduction in cardiomyocyte apoptosis and infarction area, along with a concomitant increase in angiogenesis-related factors after administration of MSC-derived sEVs using a rat MI model. The improved cardiac function was also supported by an increased left ventricular ejection fraction. It has to be noted, though, that commercially available MSCs were used for the production of sEVs in this study, indicating the need to confirm the results with extracted cells. In the same lines, Shen et al. [25] reported that administration of MSC-derived sEVs (MSC-Exo) into the injured myocardium shifted the macrophage milieu to an M2 (pro-inflammatory) phenotype, protecting against myocardial IRI. miR-21-5p was proven to contribute to this protective effect, since inhibition of miR-21-5p resulted in an upregulation of proinflammatory cytokines and an increase in the M1 macrophages. Another study by Wang et al. [26] suggested that the platelet-derived growth factor receptor-β (PDGFR-β) contributes to the protective effect of MSC-Exo by inhibiting the development of fibrosis and by promoting microvascular regeneration. In an effort to further potentiate the cardioprotective effect of MSC-EVs, Zheng et al. [27] used sEVs derived from hemin-pre-treated MSCs, as hemin had been previously shown to enhance the therapeutic efficacy of MSCs. Results showed a higher protection from cardiac senescence and fibrosis in the hemin-pre-treated, compared to the non-treated MSC-EVs. This effect was attributed to miR-183-5p, which regulates the HMGB1/ERK pathway. Of course, the contribution of other regulatory factors and miRNAs could not be excluded and requires further investigation. 

Hypoxia-conditioned bone-marrow MSC-derived sEVs, named Hypo-Exo, were also shown to protect the ischemic myocardium and promote cardiac repair by inhibiting cardiomyocyte apoptosis [41]. Interestingly, a significant enrichment of miR-125b-5p was identified in Hypo-Exo compared to the control. Knockdown of miR-125b-5p resulted in a significant increase in infract size, owing to a decreased survival of cardiomyocytes due to the loss of suppression of the proapoptotic genes p53 and BAK1. Although these results are encouraging, more work needs to be done to elucidate the possible contribution of other miRNAs in anti-apoptotic signaling.

Human-induced pluripotent stem cells (hiPSC) have also attracted a lot of interest in regenerative myocardial therapies due to their ability to differentiate to cardiomyocytes (CM), smooth muscle cells (SMC), and endothelial cells (EC), [28]. However, they carry significant limitations, such as poor retention rates, increased tumorigenesis, and ventricular arrhythmia risk, restricting their translational potential. Gao et al. [42] explored the efficacy of iPSC-CM, -SMC, and EC-derived sEVs to protect the injured myocardium in a pig MI model. sEVs were directly injected into the injured myocardium, and their protective effect was compared to that of the respective cellular compartment. Results showed comparable results in angiogenesis, LVEF, scar size, and cell apoptosis between the cellular and sEV compartment, which were significantly better than the MI group alone four weeks after infarction. However, the side-effect profile was safer within the sEV group, as no arrhythmogenic complications were noted, suggesting that iPSC-derived sEVs could offer a safe and effective alternative to cell therapy for myocardial injury. The only limitation of such therapy would be the need for a local (myocardium) injection of sEVs, since iPSC-derived sEVs are not efficiently recruited to the heart, prohibiting a systemic administration.

To overcome the limitation of potentially reduced retention time at the site of injury, various groups have successfully explored the use of hydrogels [43]. Chen et al. [44] encapsulated sEVs in shear-thinning hydrogel (STG), whereas Han et al. [45] used a peptide-based hydrogel (PGN) to load sEVs and injected them into the myocardium. In both cases, there was significant improvement in angiogenesis and hemodynamics and, most importantly, sEVs with equally successful results [46]. Other strategies, such as genetic engineering and targeting sEVs to specific sites, have also been explored with very positive results [29,47]. 

Using a murine MI model, Santoso et al. [48] injected sEVs derived from iPSC-CM at the peri-infract site to investigate their potent cardioprotective function under ischemic conditions. Matrigel was mixed with the sEVs to improve engraftment in the myocardium. A significant increase in LVEF and a greater myocardial viability were noted 2 and 4 weeks post-sEV administration compared to controls. Next, they proved that the iPSC-CM-sEVs improved the myocardial viability by salvaging the existing injured cells rather than inducing a myocardial stem cell proliferation. Autophagy was shown to mediate myocardial protection from ischemia-induced cell death via mammalian target of rapamycin (mTOR) inhibition, although mTOR singling may not be the only downstream mechanism of iPSC-CM-sEVs.

Cardiac progenitor cells (CPCs) and cardiosphere-derived cells (CDCs) have also shown promising results in adults with ischemic heart disease [49], although with significant limitations due to low retention rates and inability to monitor them in vivo [50]. However, CPC and CDC secretome, including sEVs, has been shown to promote myocardial recovery [30]. Specifically, it has been shown that CPC-sEVs protect myocardial cells from oxidative-stress-related apoptosis via sEV miR-21 by downregulation of programmed cell death 4 (PDCD4), [31]. In the same lines, CDC-sEVs have been shown to protect the infracted myocardium via sEV HSP60 [51,52]. Hypoxia-induced cardiomyocytes resulted in paracrine transfer of miRNA-30a between cardiomyocytes via sEVs, which resulted in inhibition of autophagy [32].

Zhang et al. [33], exploring the potential crosstalk between cardiomyocytes and macrophages in the context of myocardial ischemia, investigated the potential role of sEVs. Specifically, the investigators compared cardiomyocyte-derived sEVs treated under hypoxic (hypo-Exo) or normal conditions (norm-Exo) with those isolated from plasma of patients with MI (hAMI-Exo) or healthy individuals (hNor-Exo). They demonstrated a significant increase in TFG-β, inducible nitric oxide synthase (iNOS), and Arginase 1 (Arg-1) in hypo-Exo and hAMI-Exo compared to the controls. This is of interest, as TFG-β has been shown to promote myocardial repair [53]. After intramyocardial injection, a significant macrophage activation towards M2 phenotype was noted in cardiac tissue, as opposed to M1 activation, which was noted with the controls, suggesting a role in cardiac repair after MI. An increased expression of the anti-apoptotic markers B-cell lymphoma 2 (bcl-2) and bcl-2-associated X-protein (bax) was detected in the M2 macrophages, supporting their role in suppressing the oxidative stress injury of cardiomyocytes. Interestingly, 18 different miRNAs were detected in the hypo-Exo and hAMI-Exo compared to the controls, but unfortunately, the authors failed to elucidate how these regulate M2 polarization. 

**Figure 1:**
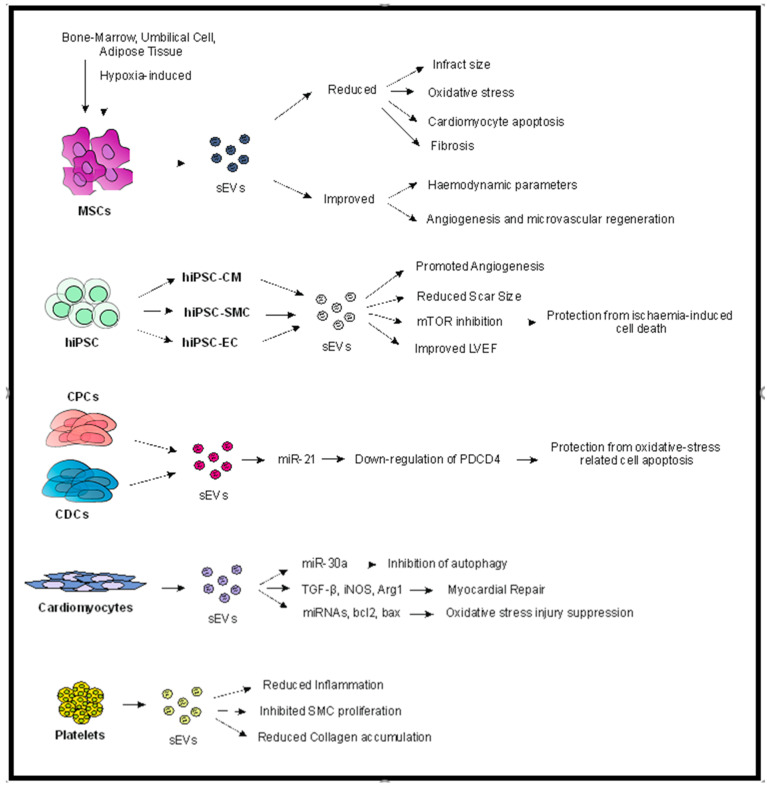
Potential contribution of sEVs in myocardial protection. As demonstrated, sEVs may be derived from various types of cells, such as MSCs (of any source), hiPSC, CPC and CDC, cardiomyocytes, and platelets. Different mechanisms of action have been proposed for the various sEV groups, although common pathways and mechanisms are identified, such as reduced cardiomyocyte apoptosis and fibrosis and increased angiogenesis, which contribute to reduced infract size and improved hemodynamic parameters. Abbreviations: sEVs: small extracellular vesicles, hiPSC: human induced-pluripotent stem cells, CM: cardiomyocytes, SMC: smooth muscle cell, EC: endothelial cell, LVEF: left ventricular ejection fraction, CPCs: cardiac progenitor cells, CDCs: cardiosphere-derived cells, PDCD4: programmed cell death 4.

Another population that has attracted attention is platelet-derived sEVs, as they may inhibit platelet activation, reduce inflammation, and attenuate smooth muscle cell proliferation, making them an attractive therapeutic candidate for coronary artery disease [34,35,36]. Li et al. [37] used platelet-derived sEVs to block IL-1 (IL1-PMs), which is known to promote an inflammatory milieu post-MI, creating abnormal cardiac remodeling. The IL1-PMs were able to neutralize IL1, protecting the cardiomyocytes from apoptosis. More importantly, they demonstrated an associated reduced collagen accumulation in the scarred myocardium, which was related to improved heart function, such as LVEF (Figure 1). However, the need for inactivated platelets prior to application, the use of DSPE-PEG (1, 2-Distearoyl-sn-glycero-3-phosphoethanolamine-Polyethylene glycol) linkers, and the failure to use an IRI model constitute significant drawbacks of the study, without eliminating their important findings.

## 4. Potential Role of sEVs in CVD Pathogenesis and Diagnosis

In the context of CVD, there is an imperative need to identify new biomarkers with potential for diagnosis and risk assessment, as the currently used markers and scores (troponin, cholesterol, low-density lipoprotein (LDL), C-reactive protein (CRP), creatinine-kinase myocardial band (CK-MB), etc.) offer little information about the stage of the disease. sEVs constitute the ideal candidate for liquid biopsy, owing to: (a) their small size, offering them the ability to circulate readily through small capillaries; (b) their ability to be produced by all living cells; (c) their cargo, reflecting that of the parental cell; and (d) their relatively easy isolation methods. In the context of cardiovascular disease, a number of studies exist supporting the role of sEVs in predicting both the development and the recurrence of ischemic heart disease (summarized in Table 2) [54].

Increased plasma levels of sEVs have been detected in concordance with cardiovascular (CV) risk factors, such as diabetes [67], hypertension, [59] smoking [68], hypercholesterolemia [69], and atherosclerosis [65]. A group of six biomarkers reflecting pathways of complement activation, lipoprotein metabolism, and platelet activation were found to be upregulated in plasma-EVs from patients with acute MI [66]. Interestingly, this panel of markers was subsequently validated in 43 patients using an antibody-based validation system, and results were concordant with the proteomics data set. Interestingly, the plasma-sEV fibrinogen was found to be significantly reduced in patients with MI, suggesting that a counteractive mechanism suppressing a post-MI hypercoagulable state may exist. Although very promising, these data need to be interpreted with caution, as they refer to a small group of patients. Larger prospective studies are required to validate the results prior to clinical translation. 

Another study by Kanhai et al. [70] investigated the predictive potential of sEVs by studying the sEV cargo in plasma from patients with a previous vascular event. Results revealed that increased levels of cystatin C, as well as SerpinF2 and CD14, in plasma sEVs were associated with increased risk of recurrent cardiovascular events and mortality. However, it has to be noted that despite the fact that the authors isolated the sEV population by ExoQuick (a well described method for sEV isolation), they failed to confirm the size and quality of the isolated population and used a broader definition of ‘microvesicles’. Furthermore, only a small number of sEV proteins were measured, and no in vitro studies were conducted to define the exact pathophysiologic relation between the sEV-proteins and atherogenesis.

Cysteine-rich protein 61 (Cyr61) has been previously shown to be significantly elevated in patients with acute coronary syndrome (ACS) and have a superior diagnostic accuracy to that of troponin T. However, its moderate sensitivity and specificity have limited its clinical use. Li et al. [55] studied the Cyr61 levels in plasma sEVs from patients with cardiovascular disease and found a significant increase in patients with acute MI compared to healthy individuals. Cyr61 levels were also shown to independently correlate with the existence of cardiovascular disease. Although the sensitivity and sensibility were higher than previously described [56], the area under the curve (AUC) was lower than that described by Deng [57], indicating that larger-scale studies are required to confirm the findings prior to translating to clinical use.

An elegant study by Saha et al. [58] provided evidence of the ability of CDC- and CPC-derived sEVs to be used as liquid biopsy. Specifically, CPC-derived sEVs were found to be significantly increased in the early post-operative period in a xenogeneic rat MI model. They also used a computational model to demonstrate that the sEV-mRNA profile can predict the state of the cardiac function, as defined by the ejection fraction, fibrosis, and angiogenesis.

Increased levels of sEV miR-1 and miR-133a have been correlated with dead cardiomyocytes [71], whereas decreased sEV miR-939-5p was identified in patients with MI, which promoted angiogenesis by enhancing NO production by the endothelium [60]. Furthermore, miR-92a-3p expression, which inhibits angiogenesis, is upregulated in endothelial cell-derived sEVs under atherosclerotic conditions [61]. A role for sEVs in the pathogenesis of atherosclerosis has also been proposed. Specifically, sEVs have been linked with the development of microcalcification in atherosclerotic plaques, as calcified sEV aggregates were identified between collagen fibers, forming calcifying structures [72]. Along the same lines, Liu et al. [73] demonstrated that sEVs released from perivascular adipose tissue carry miR-382-5p, which regulate the cholesterol efflux transporters ABCA1 and ABCG1, reducing macrophage foam cell formation. Although these results are very promising, as a means to prevent atherosclerosis, more work needs to be done to confirm the findings and possibly assess their anti-atherogenic effect in an in vivo model. Furthermore, the contribution of other miRNAs cannot be excluded, nor can that of the interaction with other pathways and factors. 

The contribution of sEVs in the pathogenesis of atherosclerosis was also supported by Xie et al. [62], who provided evidence indicating that sEVs derived from obese visceral adipose tissue (high-fat diet visceral adipose tissue-derived sEVs (HFD-VAT-Exo)) induce a pro-inflammatory M1 macrophage polarization. Furthermore, systemic administration of HFD-VAT-Exo enhanced atherosclerosis in hyperlipidemic ApoE-deficient mice, proposing a possible therapeutic target. Similarly, Barberio et al. [74], using samples from obese and non-obese adolescents, studied the possible crosstalk between adipose-tissue-derived sEVs (AT-Exo) and macrophages. Results revealed that such sEVs decreased the cholesterol efflux, irrespective of body weight. More importantly, they identified six miRNAs (miR-3129-5p, miR-20b, miR-320d, miR9-5p, miR301a-5pm and miR-155-5p) in the AT-Exo that regulate cholesterol efflux through ABCA1. Again, more work is required to confirm these results and investigate the possible interaction of such miRNAs with various cell populations (such as monocytes, platelets, endothelial cells, etc.), as well as testing various conditions under which AT-Exo regulates cholesterol efflux. 

**Figure 2:**
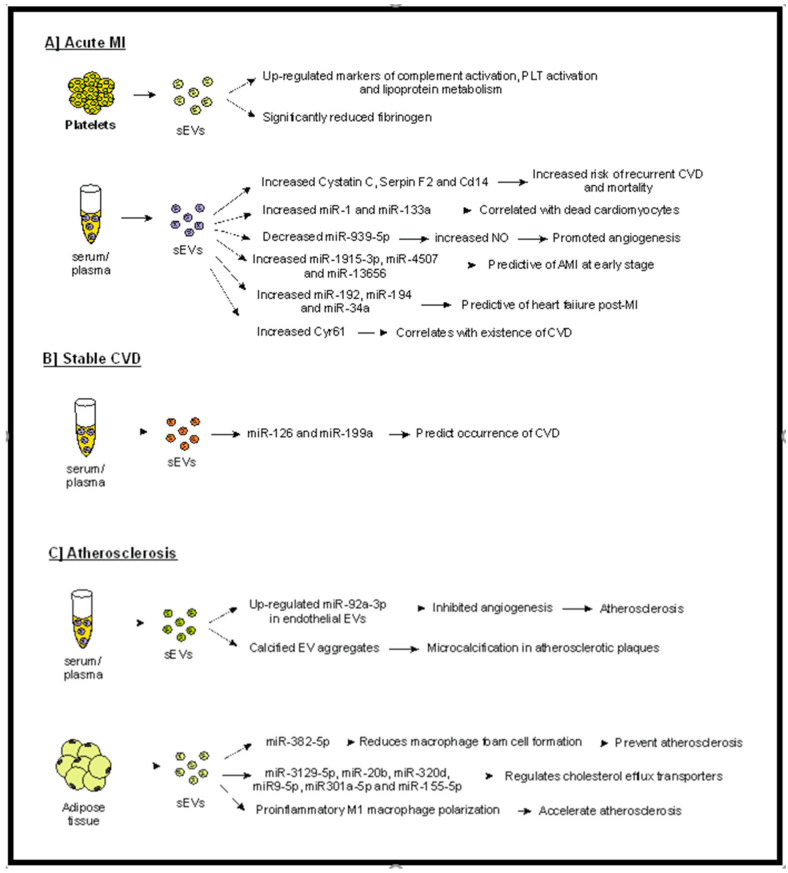
Possible predictive role of sEVs in acute MI (**A**) in stable CVD (**B**) and in the generation of atherosclerotic plaques. (**A**) sEVs isolated from serum or plasma of patients with acute MI have been shown to carry various markers, such as miRNAs, cystatin C, serpin F2, CD14, and Cyr-6, that correlate with increased morbidity (e.g., heart failure, dead cardiomyocytes, CVD). Furthermore, factors associated with bad outcomes (such as complement activation and lipoprotein metabolism) have been detected in platelet-derived sEVs in patients with acute MI. (**B**) In patients with stable CVD, certain sEV-miRNAs can predict the progression of disease. (**C**) sEVs have been proven to participate in generation of atherosclerotic plaques, whereas adipose-derived sEVs have been shown to contribute to atherosclerosis development and progression. Abbreviations: sEVs: small extracellular vesicles, PLT: platelets, AMI: acute myocardial ischemia, CVD: cardiovascular disease, Cyr-61: cysteine-rich angiogenic inducer 61.

Jansen et al. [75] demonstrated that plasma sEVs from patients with stable CVD containing miR-126 and miR-199a were able to predict the occurrence of cardiovascular events. Interestingly, these miR-126 and miR-199a-carrying sEVs were proven to be released from platelets and endothelial cells. However, only a selected number of miRNAs were studied in a relatively small number of patients, and no insight on the exact protective mechanism of miR-199a was given (Figure 2). Similarly, Su et al. [63] suggested that serum sEV miR-1915-3p, miR-4507, and miR-3656 may be predictive of acute MI at an early stage, whereas Matsumoto et al. [64] proposed sEVs as potential predictive markers for ischemic heart failure post-MI.

## 5. Conclusions

The great translational potential of the sEVs in the field of heart diseases has been well demonstrated. Preliminary in vitro and animal in vivo studies have provided enthusiastic results, and human clinical trials are awaited with great interest, as trials involving the use of sEVs in other diseases have shown both great therapeutic effect and, most importantly, no or minimal side effects [76]. 

However, despite the aforementioned promising results and the advantages of sEVs, we should note that there are still some limitations to their clinical translation. These involve the lack of a unifying protocol for the isolation, purification, and characterization of sEVs, limiting their scalability and reproducibility [77]. Furthermore, it has been shown that sEVs injected intravenously are rapidly cleared by macrophages and accumulate in organs, such as the liver, spleen, and lungs [78]. However, novel strategies have been developed to enhance their bioavailability, such as encapsulation of sEVs in hydrogels and genetic engineering of EVs, with encouraging results [43]. It is also challenging to define a ‘therapeutic sEV unit’ but to propose a mechanism of action and clarify the sEV component that is responsible for the biological action in the specific disease [79]. Overall, sEVs seem to constitute a promising tool in translational medicine and particularly in regenerative medicine when referring to CVD, although clinical trials are required to prove their efficacy and safety.

## Figures and Tables

**Table 1:** Studies demonstrating the various sEV sources and the respective cardioprotective effect and mechanism of action.

Donor Cell	Functional Component	Functional Outcome	References
hESC	TGF-β signalling	Reduction in oxidative stress, reduced cell apoptosis	[22]
MSC	Increase in angiogenesis-related factors miR-21-5p PDGFR-β miR-183-5p, regulating HMGB1/ERK pathway	Reduction in cardiomyocyte apoptosis, M2 polarizationInhibition of fibrosis and microvascular regeneration Inhibition of cardiac senescence and fibrosis	[23] [24] [25] [26]
Hypo-BM-MSC	Enrichment of miR-125b-5p and suppression of p53 and BAK1	Inhibition of cardiomyocyte apoptosis and promotion of cardiac repair	[27]
iPSC-CM, -SMC and EC	CXCR4, miR-100, miR-21-5p, miR-199a-3p mTOR inhibition	Improved angiogenesis and cardiac repair Improved myocardial viability	[28] [29]
CPC	sEV miR-21, downregulating PDCD4	Promotes myocardial recovery	[30]
CDC	sEV HSP60	Inhibition of cardiac cell apoptosis and fibrosis	[31]
Hypoxia-induced cardiomyocytes	miRNA-30a	Inhibition of autophagy	[32]
Cardiomyocytes	Increased TGF-β, iNOS, Arg1 Increased bax and bcl2 M2 polarization	Myocardial repair Oxidative stress injury suppression Anti-inflammatory environment	[33]
Platelets	CD36 downregulation miR-223, downregulating ICAM-1 via NF-kB miR-223, miR-339, miR-21 regulating PDGFRβ IL-1 inhibition	Inhibition of PLT activation Reduction in endothelial inflammation Inhibition of VSMCs proliferation Inhibition of cardiomyocyte apoptosis	[34] [35] [36] [37]

Abbreviations: sEV: small extracellular vesicles, hESC: human embryonic stem cells, MSC: mesenchymal stem cells, Hypo-BM-MSC: hypoxia-conditioned bone-marrow MSC, iPSC-CM, -SMC and EC: Human induced pluripotent stem cell-cardiomyocytes, -smooth muscle cells, and -endothelial cells, LVEF: left ventricular ejection function, CPC: cardiac progenitor cells, PDCD4: programmed cell death 4, CDC: cardiosphere-derived cells, TGF-β: transforming growth factor beta, iNOS: inducible nitric oxide synthase, Arg1: Arginase-1, PLT: platelets, ICAM-1: intercellular adhesion molecule-1, PDGFRβ: platelet-derived growth factor receptor-beta, VSMC: vascular smooth muscle cells.

**Table 2:** Predictive potential of plasma sEVs.

EV Source	Biomarker	Clinical Prediction/Association	References
Plasma	Total sEVs, platelet-, monocyte- and endothelium-sEVs	Diabetes, hypertension, smoking, hypercholesterolemia	[55,56,57,58]
Plasma	C1QA, C5, APOD, APOCC3, GP1BA, PPBP Decreased sEV fibrinogen	Coronary artery disease	[59]
Plasma from patients with previous CVD	sEV Cystatin C, Serpin F2 and CD14	Increased risk of recurrence and death	[60]
Patients with ACS	sEV Cyr61	Predicts for the existence of CVD	[61]
Plasma	Increased sEV miR-1, miR-133a and miR-92a-3p Decreased sEV miR-939-5p	Correlated with dead cardiomyocytes and inhibition of angiogenesis Promotes angiogenesis	[62]
Perivascular AT	sEV miR-382-5p	Reduces macrophage foam cell formation	[63]
HFD-VAT	sEV miR-3129-5p, miR-20b, miR-320d, miR9-5p, miR301a-5p and miR-155-5p	Regulates cholesterol efflux	[64]
Plasma from patients with stable CVD	sEV miR-126 and miR-199a sEV miR-1915-3p, miR-4507	Predicts for the occurrence of CVD Predictive of acute MI	[65] [66]

Abbreviations: sEVs: small extracellular vesicles, C1QA: complement C1q subcomponent subunit A, C5: complement C5, APOD: apolipoprotein D, APOCC3: apolipoprotein C-III, GP1BA: platelet glycoprotein Ib alpha chain, PPBP: platelet basic protein, MI: myocardial ischemia, CVD: cardiovascular disease, ACS: acute coronary syndrome, Cyr61: cysteine-rich 61, AT: adipose tissue, HFD-VAT: high-fat diet visceral adipose tissue.
